# Factors predicting the need for hemorrhage control intervention in patients with blunt pelvic trauma: a retrospective study

**DOI:** 10.1186/s12893-018-0438-8

**Published:** 2018-11-16

**Authors:** Myoung Jun Kim, Jae Gil Lee, Seung Hwan Lee

**Affiliations:** Department of Surgery, Yonsei University College of Medicine, 50-1 Yonsei-ro, Seodaemun-gu, Seoul, 03722 Republic of Korea

**Keywords:** Pelvis, fracture, hemorrhage control

## Abstract

**Background:**

Blunt pelvic injuries are often associated with pelvic fractures and injuries to the rectum and genitourinary tract. Pelvic fractures can lead to life-threatening hemorrhage, which is a common cause of morbidity and mortality in trauma. Thus, early identification of patients with pelvic fractures at risk severe bleeding requiring urgent hemorrhage control is crucial. This study aimed to investigate early factors predicting the need for hemorrhage control in blunt pelvic trauma.

**Methods:**

The medical records of 1760 trauma patients were reviewed retrospectively between January 2013 and June 2018. We enrolled 187 patients with pelvic fracture due to blunt trauma who were older than 15 years. The pelvic fracture pattern was classified according to the Orthopedic Trauma Association/Arbeitsgemeinschaft fur Osteosynthesefragen (OTA/AO) classification. A multivariate logistic regression model was used to determine independent predictors of the need for pelvic hemorrhage control intervention.

**Results:**

The most common pelvic fracture pattern was type A (54.5%), followed by types B (36.9%) and C (8.6%). Of 187 patients, 48 (25.7%) required pelvic hemorrhage control intervention. Hemorrhage control interventions were most frequently performed in patients with type B fractures (54.2%). Multivariate logistic regression analysis revealed that type B (odds ratio [OR] = 4.024, 95% confidence interval [CI] = 1.666–9.720, *p* = 0.002) and C (OR = 7.077, 95% CI = 1.781–28.129, *p* = 0.005) fracture patterns, decreased body temperature (OR = 2.275, 95% CI = 0.134–0.567, *p* < 0.001), and elevated serum lactate level (OR = 1.234, 95% CI = 1.061–1.435, *p* = 0.006) were factors predicting the need for hemorrhage control intervention in patients with blunt pelvic trauma.

**Conclusion:**

Patients with type B and C fracture patterns on the OTA/AO classification, hypothermia, or an elevated serum lactate level are at risk for bleeding and require pelvic hemorrhage control intervention.

**Electronic supplementary material:**

The online version of this article (10.1186/s12893-018-0438-8) contains supplementary material, which is available to authorized users.

## Background

Pelvic injuries occur frequently, amounting to almost 9% of all blunt trauma patients [1]. Blunt pelvic injuries from high-energy mechanisms such as a fall from a height or road traffic collision are often associated with pelvic fractures and injuries to the rectum and genitourinary tract [1–4]. The seriousness of blunt pelvic fractures lies in the possible occurrence of retroperitoneal hematomas and hemorrhagic shock [5, 6]. Most pelvic hemorrhage occurs from venous and fracture sites (85%) [7, 8]. However, in the hemodynamically unstable patient with severe pelvic injury, arterial bleeding is frequent [4, 8]. The overall mortality rates of patients with pelvic ring fractures range from 8% to 13.5% [1, 9–11]. Pelvic bone fractures with hemodynamic instability are associated with a higher incidence of pelvic vascular injury and hemorrhage, and the mortality rates are reported to be 30%-57% [10, 12, 13].

The recent evolution of rapid pelvic stabilization by external fixation or pelvic binding, and of hemostasis by angiographic embolization, resuscitative endovascular balloon occlusion, or preperitoneal pelvic packing has significantly decreased the mortality rates in devastating pelvic injuries [14–20]. However, early detection of bleeding is not easy in blunt pelvic fractures. Furthermore, in blunt pelvic trauma with hemodynamic instability, it is difficult to achieve adequate hemostasis due to rapid exsanguination.

Therefore, early recognition of bleeding is important because it may increase the success rate of non-surgical treatment, such as angioembolization, and even if surgery is indicated, early surgery has better prognosis. If hemorrhage and coagulopathy progress significantly, no treatment can be expected to produce good clinical outcomes [18, 19, 21]. Thus, the purpose of this study was to investigate early factors predicting the need for hemorrhage control intervention in patients with blunt pelvic trauma.

## Methods

### Patient enrollment and data collection

**Fig. 1:**
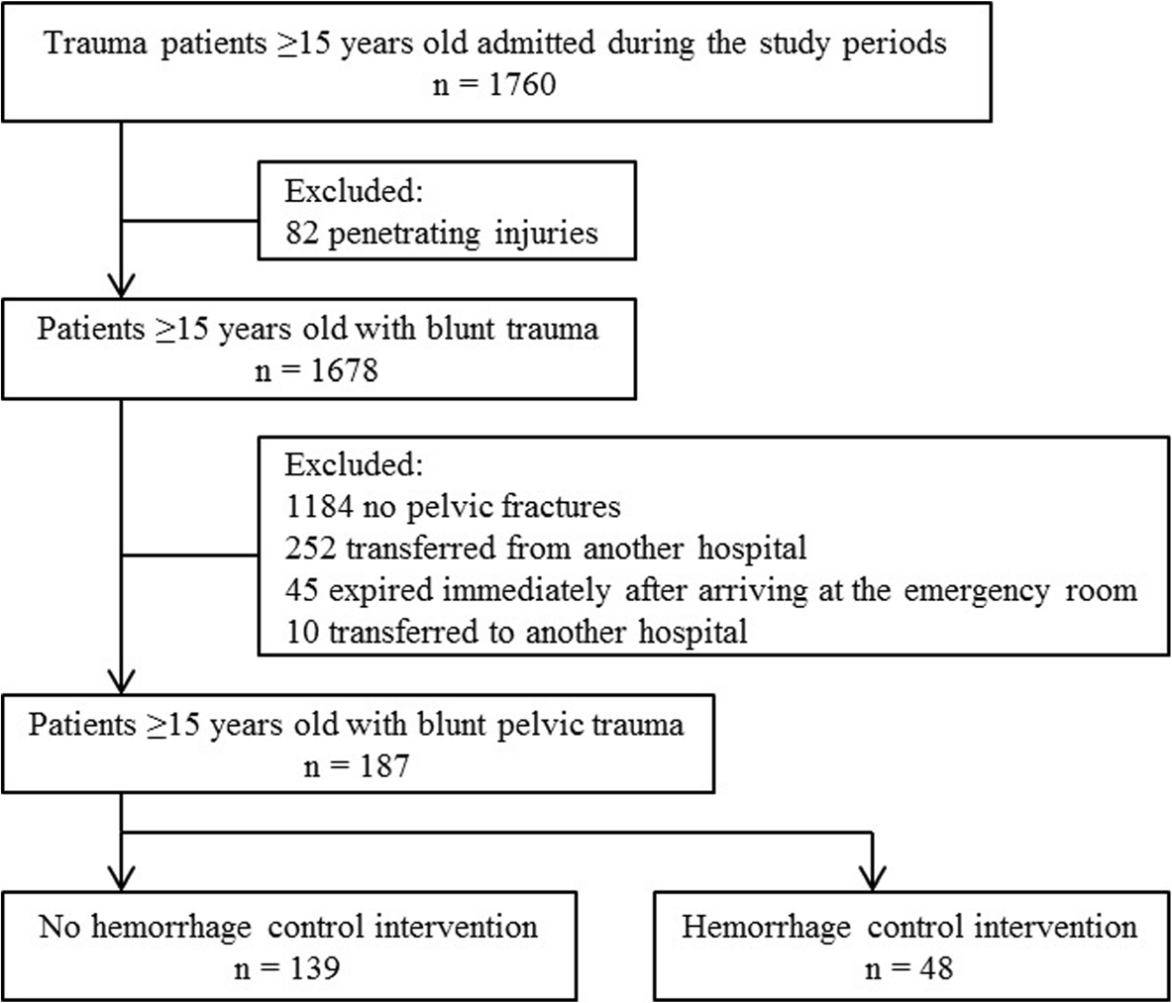
Flow diagram of patients selected for analysis

We conducted a retrospective observational study at a single center in an urban setting in Seoul, South Korea, from January 2013 to June 2018. Of 1760 trauma patients ≥15 years, patients with penetrating injuries (n = 82) were excluded. In patients with blunt trauma (n = 1678), we also those with no pelvic fractures (n = 1184), those who had been managed or evaluated at other hospitals (n = 252), those who died within minutes after arrival in the emergency room (n = 45), and/or those who were referred to other hospitals (n = 10). Therefore, the study was conducted with a total of 187 patients (Fig. 1). Patients were divided into those who did not undergo hemorrhage control intervention and those who did. Hemorrhage control intervention was defined as angioembolization, external fixation, or preperitoneal pelvic packing.

### Clinical variables

We analyzed the sex, age, injury mechanism, vital signs, Glasgow Coma Scale (GCS) score, current anticoagulant use, Abbreviated Injury Scale (AIS) score, Injury Severity Score (ISS), Revised Trauma Score (RTS), Trauma and Injury Severity Score (TRISS), Acute Physiology and Chronic Health Evaluation (APACHE) II score, and arterial blood values (pH, base excess, and lactate level). In addition, pelvic radiography of each patient was identified, and patterns of pelvic fracture were classified as types A, B, and C according to the Orthopedic Trauma Association/Arbeitsgemeinschaft fur Osteosynthesefragen (OTA/AO) classification (Table 1). The classification was based on the findings of trauma surgeons and orthopedic surgeons, which were additionally confirmed by surgical and radiological records.

**Table 1:** OTA/AO classification of pelvic fracture

Type	Description
Type A: Stable – posterior arch is intact	A1: Fracture does not involve the pelvic ring (avulsion fracture or fracture of the iliac wing) - A1.1: Iliac spine - A1.2: Iliac crest - A1.3: Ischial tuberosity
	A2: Stable or minimally displaced fracture of the pelvic ring - A2.1: Iliac wing fractures - A2.2: Unilateral fracture of anterior arch - A2.3: Bifocal fracture of anterior arch
	A3: Transverse fracture of the sacrum - A3.1: Sacrococcygeal dislocation - A3.2: Sacrum undisplaced - A3.3: Sacrum displaced
Type B: Rotationally unstable, vertically stable – incomplete disruption of the posterior arch	B1: Open book injury (external rotation) - B1.1: Sacroiliac joint, anterior disruption - B1.2: Sacral fracture
	B2: Lateral compression injury (internal rotation) - B2.1: Anterior compression fracture, sacrum - B2.2: Partial sacroiliac joint fracture, subluxation - B2.3: Incomplete posterior iliac fracture
	B3: Bilateral type B fracture - B3.1: Bilateral open book fracture - B3.2: Open book fracture and lateral compression - B3.3: Bilateral lateral compression
Type C: Rotationally and vertically unstable – complete disruption of the posterior arch	C1: Unilateral fracture - C1.1: Fracture of the iliac bone - C1.2: Sacroiliac dislocation and/or fracture dislocation - C1.3: Sacral fracture
	C2: Bilateral fracture with one side type B fracture (rotationally unstable) and one side type C fracture (vertically unstable)
	C3: Bilateral fracture with both sides type C fracture (both sides completely unstable)

*OTA/AO* Orthopedic Trauma Association/Arbeitsgemeinschaft fur Osteosynthesefragen.

### Statistical analysis

Statistical analysis for investigated items was performed using SPSS® Statistics 23.0 (IBM, Armonk, NY, USA). Categorical data are presented as numbers (%), and they were compared using the chi-square or Fisher’s exact tests. Continuous variables are expressed as mean ± standard deviation or medians (the 25th and 75th quantiles), and the data were compared between groups using the Student t-test or Mann-Whitney *U* test. Factors found to be significantly associated with the need for hemorrhage control intervention on univariate analysis were included in the multivariate analysis. Logistic regression modeling was performed using the maximum likelihood method and backward stepwise selection. Goodness of fit was assessed using the Hosmer-Lemeshow test. The odds ratios (ORs) are given with 95% confidence intervals (CIs). A *p* value <0.05 was considered statistically significant.

## Results

### Patients

The baseline characteristics of patients are listed in Table 2. Of the 187 patients, 48 underwent hemorrhage control intervention and 139 did not undergo hemorrhage control intervention. The sex ratio was 125:62 (66.8%:33.2%, male/female). There was no significant difference between the hemorrhage control and non-hemorrhage control intervention groups (*p* = 0.290). There was no significant difference between the two groups in the use of anticoagulants (*p* = 0.530). When classified according to the injury mechanism, pedestrian trauma injuries caused by motor vehicle accidents were the most frequent in both groups, followed by falls, which had the second highest frequency. However, there was no statistically significant difference between the groups that received hemorrhage control intervention and those that did not (*p* = 0.497).

**Table 2:** Baseline characteristics of patients

	No hemorrhage control intervention (n = 139)	Hemorrhagic control intervention (n = 48)	*p* Value
Age (years)	49.1 ± 19.9	53.9 ± 20.5	0.154
Sex			0.290
Male	96 (69.1)	29 (60.4)	
Female	43 (30.9)	19 (39.6)	
Anticoagulant use	8 (5.8%)	4 (8.3%)	0.530
Injury mechanism			0.497
MVA (pedestrian)	51 (36.7)	23 (47.9)	
MVA (passenger)	11 (7.9)	2 (4.2)	
Motorcycle accidents	23 (16.5)	5 (10.4)	
Falls	51 (36.7)	16 (33.3)	
Others	3 (2.2)	2 (4.2)	
AIS			
Head and neck	1.0 (0.0, 2.0)	2.0 (0.0, 3.0)	0.264
Face	0.0 (0.0, 1.0)	0.0 (0.0, 1.0)	0.539
Chest	0.0 (0.0, 3.0)	2.0 (0.0, 3.0)	0.049
Abdomen	0.0 (0.0, 2.0)	2.5 (0.0, 3.0)	<0.001
Extremities	2.0 (2.0, 3.0)	3.5 (2.0, 4.0)	<0.001
External	1.0 (1.0, 1.0)	1.0 (1.0, 1.0)	0.754
ISS	17.4 ± 11.2	30.1 ± 13.4	<0.001
RTS	7.092 ± 1.482	6.123 ± 2.089	0.004
TRISS (%)	87.09 ± 23.07	68.67 ± 32.13	0.001
APACHE II	16.1 ± 8.6	24.6 ± 10.7	<0.001
In-hospital mortality	9 (6.5)	17 (35.4)	<0.001

Values are presented as mean ± SD or n (%).

*MVA* motor vehicle accident, *AIS* Abbreviated Injury Scale, *ISS* Injury Severity Score, *RTS* Revised Trauma Score, *TRISS* Trauma and Injury Severity Score, *APACHE* Acute Physiology and Chronic Health Evaluation

When the AIS values of the two groups were compared, both systems, such as the abdomen (*p* < 0.001) and the extremity (*p* < 0.001), showed a significant difference. However, there were no significant differences in other systems. Furthermore, there were significant differences in the ISS (*p* < 0.001), RTS (*p* = 0.004), TRISS (*p* = 0.001), and APACHE II score (*p* < 0.001) between the two groups.

### Clinical variables comparison

The vital signs and laboratory variables of patients are shown in Table 3. Systolic blood pressure (*p* = 0.008) and body temperature (*p* < 0.001) in the hemorrhage control intervention group were significantly lower than those in the non-hemorrhage control intervention group. In the arterial gas analysis, the base excess (*p* = 0.001) was significantly lower in the hemorrhage control intervention group than those in the non-hemorrhage control intervention group, and lactate (*p* < 0.001) was significantly higher.

**Table 3:** Comparison of clinical parameters between two groups

	No hemorrhage control intervention (n = 139)	Hemorrhagic control intervention (n = 48)	*p* Value
Vital sign			
SBP	118.8 ± 33.7	98.8 ± 46.2	0.008
HR	88.7 ± 23.3	96.1 ± 31.1	0.132
RR	18.7 ± 4.5	17.3 ± 7.2	0.223
BT	36.4 ± 0.5	35.9 ± 0.7	< 0.001
GCS	12.9 ± 4.0	11.4 ± 4.8	0.057
ABGA			
pH	7.38 ± 0.08	7.33 ± 0.16	0.057
BE	-3.48 ± 3.30	-6.71 ± 5.79	0.001
Lactate	2.99 ± 1.89	5.42 ± 4.26	< 0.001

Values are presented as mean ± SD.

*ABGA* arterial blood gas analysis, *SBP* systolic blood pressure, *HR* heart rate, *RR* respiration rate, *BT* body temperature, *GCS* Glasgow Coma Scale, *BE* base excess

### Comparison between two groups according to pelvic fracture pattern

According to the classification of posterior pelvic ring stability based on OTA/AO, type B with partial instability was the most common (n = 26, 54.2%) in the group with hemorrhage control intervention, followed by types A and C. Type A (n = 91, 65.5%) was the most common in the non-hemorrhage control intervention group, followed by types B and C. Overall, there was significant difference (*p* < 0.001) between the groups with and without hemorrhage control intervention according to the OTA/AO classification (Table 4).

**Table 4:** Comparison via pelvic fracture pattern of patients between two groups (OTA/AO)

	No hemorrhage control intervention (n = 139)	Hemorrhagic control intervention (n = 48)	*p* Value
Pelvic fracture pattern			<0.001
A	91 (65.5)	11 (22.9)	
B	43 (30.9)	26 (54.2)	
C	5 (3.6)	11 (22.9)	

*OTA/AO* Orthopedic Trauma Association/Arbeitsgemeinschaft fur Osteosynthesefragen

### Logistic regression analysis for predictors of hemorrhage control intervention

The results of the univariate and multivariate regression analysis models are shown in Table 5. As a result, in the pelvic bone fracture pattern according to OTA/AO classification, types B (OR = 4.024, 95% CI = 1.666–9.720, *p* = 0.002) and C (OR = 7.077, 95% CI = 1.781–28.129, *p* = 0.005) were identified as predictors of hemorrhage control intervention. Among the clinical parameters, body temperature (OR = 0.275, 95% CI = 0.134–0.567, *p* < 0.001) and lactate (OR = 1.234, 95% CI = 1.061–1.435, *p* = 0.006) were identified as predictors. Furthermore, although univariate analysis revealed that the two groups had no significant differences in the use of anticoagulants, we additionally conducted a multivariable logistic regression analysis including “anticoagulant use” as covariate, given its proven clinical significance. There was no difference in the results (Additional file 1).

**Table 5:** Multivariable regression analysis according to OTA/AO classification

Characteristics	Univariate analysis		Multivariate analysis	
	OR (95% CI)	*p* Value	OR (95% CI)	*p* Value
Pelvic fracture pattern				
A	Ref.			
B	5.002 (2.264–11.052)	< 0.001	4.024 (1.666–9.720)	0.002
C	18.200 (5.328–62.166)	< 0.001	7.077 (1.781–28.129)	0.005
SBP	0.986 (0.977–0.995)	0.003		
BT	0.205 (0.104–0.401)	< 0.001	0.275 (0.134–0.567)	< 0.001
Base excess	0.847 (0.780–0.920)	< 0.001		
Lactate	1.336 (1.162–1.536)	< 0.001	1.234 (1.061–1.435)	0.006

*SBP* systolic blood pressure, *BT* body temperature, *OR* odds ratio, *CI* confidence interval, *OTA/AO* Orthopedic Trauma Association/Arbeitsgemeinschaft fur Osteosynthesefragen

## Discussion

Blunt pelvic injuries from high-energy mechanisms are often associated with pelvic fractures and injuries to the rectum and genitourinary tract [1–4]. The seriousness of blunt pelvic fractures lies in the possible occurrence of retroperitoneal hematomas and hemorrhagic shock [5, 6]. Unstable pelvic fractures are associated with massive hemorrhage [22], which is the leading cause of death in patients with major pelvic fractures [23, 24]. In the present study, patients with pelvic bleeding had significantly higher in-hospital mortality rates than those without pelvic bleeding. Moreover, among patients with pelvic bone fractures, trauma-related severity scores such as the GCS, ISS, RTS, and TRISS were significantly higher in the hemorrhage control intervention group than in the non-hemorrhage control intervention group. Therefore, trauma patients in need of emergent intervention or surgery for ongoing hemorrhage have increased chances of survival if the elapsed time between traumatic injury and bleeding control intervention is minimized [5, 10, 16, 25].

The recent evolution of rapid pelvic stabilization by external fixation or pelvic binding and of hemostasis by angiographic embolization, resuscitative endovascular balloon occlusion, or preperitoneal pelvic packing has significantly decreased the mortality rates of devastating pelvic injuries. However, early detection of bleeding is not easy in blunt pelvic fractures [14–20]. Furthermore, despite ongoing bleeding in a severely injured patient arriving at a hospital, the vital signs of the patient may not show typical changes in the immediate and early periods after injury [26, 27]. In blunt pelvic trauma with hemodynamic instability, it is difficult to achieve adequate hemostasis due to rapid exsanguination. Therefore, early and quick prediction of the need for hemorrhage control interventions for pelvic injuries is important.

In the present study, type B and C fractures according to the OTA/AO classification were revealed as independent factors predicting the need for hemorrhage control intervention in patients with blunt pelvic trauma. Type B and C fractures show pelvic bone fracture patterns including posterior pelvic ring instability. Type B fracture is a result of rotational forces that cause partial disruption of the posterior sacroiliac complex [28, 29]. Complete disruption of the posterior complex occurs in type C fractures, which are both rotationally and vertically unstable [28, 29]. In this study, the need for early hemorrhage control interventions was 4 and 7 times higher for type B and C fractures than for type A fractures, respectively.

Although patients with high-grade pelvic ring injuries may not have significant bleeding, the bleeding risk generally increases with the degree of instability of the posterior pelvic ring [30, 31]. Manson et al. [24] reported that transfusion requirements and mortality were significantly higher in the posterior ring instability pattern than in the pelvic bone fracture pattern *without* involvement of the *posterior* structures, and this suggests that stretching and tearing of soft tissues, like artery and vein, around the posterior pelvic ring showed greater hemorrhagic instability in lateral compression III, anterior-posterior compression III, and vertical shear. Costantini et al. [32] similarly concluded that there is a higher need for hemorrhage control intervention in the posterior pelvic ring instability patterns, such as anterior-posterior compression III or open pelvic fracture. In the current guidelines, markers of pelvic hemorrhage also include anterior-posterior and vertical shear deformations on standard roentgenograms [33–35].

In the present study, patterns of pelvic fracture were evaluated with pelvic radiography. To date, CT has replaced radiography in classifying pelvic fractures [35]. Contrast-enhanced CT also helps diagnose pelvic hematoma and active extravasation of contrast [7]. Multidetector CT has short acquisition times and allows for rapid identification and assessment of pelvic hemorrhage [36]. However, CT cannot be performed for all patients and is dependent on the situation, such as hemodynamic instability or absence of resources in each institution [36]. Although evaluation of the sacrum and sacroiliac joints is sometimes limited on a portable anteroposterior pelvic radiograph, pelvic radiography is one of the tools that can easily and quickly reveal the pelvic bone fracture pattern, and it is generally performed as an initial examination in the trauma bay [37, 38]. Furthermore, pelvic radiography in hemodynamically unstable patients helps in identifying life-threatening pelvic ring injuries [39].

The patterns of pelvic fracture were classified as types A, B, and C using the OTA/AO classification. The OTA/AO classification was based on fracture stability, especially the stability of the posterior lesion [28, 40–42]. Unstable pelvic fractures are more frequently associated with hemorrhage [4, 8]. The OTA/TA classification is easier to use in classifying patterns of pelvic fracture through pelvic radiography than the Young-Burgess classification, which is based on mechanism of injury [38, 42]. Furthermore, the Young-Burgess classification scheme for pelvic ring injury basically cannot be used to guide transfusion requirements and the need for angiography and embolization in individual cases [30].

In the multivariable regression analysis, the body temperature was significantly low in the hemorrhage control intervention group. In other words, hypothermia was a predictor of the need for hemorrhage control intervention. Hypothermia is common in trauma victims and is associated with an increased risk of severe bleeding and increased mortality [43, 44]. In the study of Gentilello et al. [45], the group of trauma patients with a mean body temperature of 34.5°C showed a mortality of 100% when they failed to be rewarmed to 36°C. Therefore, warming and euthermia in a trauma patient with pelvic bone fracture are crucial.

The current guidelines recommend either serum lactate or base deficit measurements as sensitive tests to estimate and monitor the extent of bleeding and shock [46–49]. Additionally, serial measurement of these parameters can be used to monitor the response to therapy [48]. The amount of lactate produced by anaerobic glycolysis is an indirect marker of oxygen debt, tissue hypoperfusion, and severity of hemorrhagic shock [47, 50]. Similarly, base deficit values derived from arterial blood gas analysis provide an indirect estimation of global tissue acidosis due to impaired perfusion [50, 51]. Moreover, the lactate and base deficit have been mentioned in many studies as predictive values related to bleeding in pelvic bone fractures in trauma patients [52–55]. In this study, serum lactate was identified as an independent predictor of the need for hemorrhage control intervention. In other previous studies, both initial serum lactate and lactate clearance after 6 hours were identified as independent risk factors for mortality in trauma patients [56]. In addition, an increased serum lactate level is associated with massive hemorrhage in pelvic ring fractures [57], and the serum lactate level measured in the pre-hospital period was found to be associated with the clinical outcome in trauma patients [58].

We additionally conducted a multivariable logistic regression analysis including “anticoagulant use” as potential predictor. It is indisputable that anticoagulants may worsen bleeding in a trauma patient. However, despite the clinical significance of anticoagulants, the results have not changed whether anticoagulant use is included or not in the multivariable logistic regression analysis. Therefore, our results should be carefully interpreted and used strictly in a wider context of the patient’s clinical condition, clinical setting, and individual included factors.

There are several limitations to the present study. First, it is a retrospective study. Second, it may be difficult to generalize the results of this study, as it is a single-center study. Third, the statistical power is insufficient because of the small number of subjects. Therefore, multicenter studies are needed to overcome these limitations.

## Conclusion

OTA/AO type B and C fractures, hypothermia, and increased lactate level are independent factors predicting the need for hemorrhage control intervention in patients with blunt pelvic traumas. Type B and C fractures are more likely to be associated with vascular injuries than are type A fractures. Hypothermia is well known to worsen coagulopathy. Lactate is a marker of systemic tissue perfusion and is elevated in cases of hypoperfusion of the tissues, such as hemorrhagic shock. These three factors may reflect the severity and occurrence of pelvic bleeding in patients with blunt pelvic trauma. Therefore, the predictors can be helpful in making decisions about management of pelvic bone fractures with hemorrhage.

## Additional file

Additional file 1: Multivariable regression analysis according to OTA/AO classification. (DOCX 14 kb)

## Abbreviations

AIS: Abbreviated Injury Scale
APACHE: Acute Physiology and Chronic Health Evaluation
BE: Base excess
BT: Body temperature
GCS: Glasgow Coma Scale
HR: Heart rate
ISS: Injury Severity Score
LOS: Length of stay
MV: Mechanical ventilation
MVA: Motor vehicle accident
RR: Respiration rate
RTS: Revised Trauma Score
SBP: Systolic blood pressure
TRISS: Trauma and Injury Severity Score Injury Severity Score
