# Supplementary material for: Factors predicting the need for hemorrhage control intervention in patients with blunt pelvic trauma: a retrospective study
Source: BMC Surg. 2018 Nov 16;18:101. doi: 10.1186/s12893-018-0438-8 (PMC6240179; doi:10.1186/s12893-018-0438-8)
Supplement: Supplementary file 1 — Multivariable regression analysis according to OTA/AO classification. (DOCX 14 kb) [file 12893_2018_438_MOESM1_ESM.docx]

Additional file 1. Multivariable regression analysis according to OTA/AO classification

| Characteristics | Univariate analysis | | Multivariate analysis | |
| --- | --- | --- | --- | --- |
|  | OR (95% CI) | *p* Value | OR (95% CI) | *p* Value |
| Pelvic fracture pattern |  |  |  |  |
| A | Ref. |  |  |  |
| B | 5.002 (2.264–11.052) | <0.001 | 4.753 (1.875–12.048) | 0.001 |
| C | 18.200 (5.328–62.166) | <0.001 | 7.907 (1.917–32.614) | 0.004 |
| SBP | 0.986 (0.977–0.995) | 0.003 |  |  |
| BT | 0.205 (0.104–0.401) | <0.001 | 0.284 (0.138–0.585) | 0.001 |
| Base excess | 0.847 (0.780–0.920) | <0.001 |  |  |
| Lactate | 1.336 (1.162–1.536) | <0.001 | 1.236 (1.064–1.436) | 0.006 |
| Anticoagulant use | 1.489(0.427–5.185) | 0.532 | 4.302 (0.811–22.815 | 0.087 |

SBP = systolic blood pressure, BT = body temperature, OR = odds ratio, CI = confidence interval, OTA/AO the Orthopedic Trauma Association/Arbeitsgemeinschaft fur Osteosynthesefragen.
